# Effect of Post Thermal Annealing on the Optical Properties of InP/ZnS Quantum Dot Films

**DOI:** 10.1186/s11671-018-2784-y

**Published:** 2018-11-20

**Authors:** Bowen Zhang, Zhipeng Wei, Xinwei Wang, Xuan Fang, Dengkui Wang, Xian Gao, Dan Fang, Xiaohua Wang, Rui Chen

**Affiliations:** 1 State Key Laboratory of High Power Semiconductor Laser, School of Science, Changchun University of Science and Technology, 7089 Wei-Xing Road, Changchun, 130022 China; 2 State Key Laboratory of High Power Semiconductor Laser, School of Materials Science and Engineering, Changchun University of Science and Technology, 7089 Wei-Xing Road, Changchun, 130022 China; 3 Department of Electrical and Electronic Engineering, Southern University of Science and Technology, Shenzhen, 518055 Guangdong China

**Keywords:** InP/ZnS QD film, Thermal annealing, Optical properties

## Abstract

The enhancement of optical properties via thermal annealing on InP/ZnS core/shell quantum dot (QD) film was investigated in this work. The increase of emission intensities of the QD films was observed after thermal annealing at 180 °C for 5 min. Through temperature dependence photoluminescence (TDPL) and power dependence photoluminescence (PL) measurement, the peak located at the low-energy shoulder was confirmed to be localized state emission and the high energy one comes from free-carrier emission. Moreover, from the TDPL spectra of the sample annealed at 180 °C for 5 min, the full width at half maximum (FWHM) of localization state emission was nearly the same before which is 250 K and then decreased with increasing temperature. However, the FWHM was decreased significantly when temperature increased in the untreated sample. We conclude that the escape of localization states with increasing temperature contributes to this anomaly phenomenon. Our studies have significance on the application of QDs in electroluminescence devices and down-conversion light-emitting devices.

## Introduction

Colloidal quantum dots (QDs) have various applications such as displays [1], spectrometers [2], sensing [3], light-emitting diodes [4], laser [5], photoelectrochemicals [6, 7], and biolabeling [8]. InP-based QDs appear to be an ideal candidate for Cd-based QDs due to their similar band gap to CdSe, band gap tenability which covers the entire visible range, and the reduced toxicity [9]. It is reported that the synthesized InP QDs always have a larger size distribution, and the full width at half maximum (FWHM) of the photoluminescence (PL) spectra of InP-based QDs usually lie in the range of 50–100 nm. This value is significantly larger than the Cd-based QDs, where the typical FWHM is around 20–30 nm. Considering the difficult synthesis and the larger size distribution of InP-based QDs, it still needs a lot of work to do for researchers.

At the same time, due to the surface traps, dangling bonds, stacking faults in the crystal, and a high activation barrier for the trapping centers, the PL quantum yield (QY) of InP QDs is relatively low (< 1%) [10]. Strategies to enhance the emission include chemical modification the particle surface [11, 12], or epitaxial growth of a shell of larger band gap semiconductors [13–15]. These strategies aim to reduce non-radiative recombination centers by surface passivation. Beside the above treatments, researchers also use thermal annealing to improve the crystalline properties of materials. It is known that thermal treatment can remove the organic surfactants from the surface of QDs to reduce the distance and consequently increase the electronic coupling between the QDs [16, 17]. Post thermal treatment processes have significantly influenced the optical and electrical properties of the QDs and improved the performance of QD-based optoelectronic devices. And it is essential to understand the effect of thermal annealing on the carriers’ recombination processes inside core/shell QDs for better device performance.

Here we fabricated InP/ZnS core-shell QD films by spin coating. QDs were spun onto the Si substrate to form a solid film. The films were annealed at different temperatures. We measured the PL spectra for these samples at 300 K, and it is found that only the sample annealed at 180 °C shows enhanced light emission. Temperature- and power-dependent PL measurements were carried out, and the untreated sample was compared with the sample annealed at 180 °C. Based on the experimental results, the origin of the peak of QD films and the effect of annealing have been discussed in detail.

## Methods

InP/ZnS nanocrystals are synthesized based on protocols published on other papers [8, 9]. Two hundred milligrams (0.45 mmol) of indium (III) chloride and 122 mg (2.2 mmol) of zinc (II) chloride are mixed in 5 mL of oleylamine which is a coordinating solvent. The reaction mixture is stirred and degassed at 100 °C for half an hour and then heated to 220 °C under inert atmosphere. Upon reaching 220 °C, a volume of 0.25 mL oftris(dimethylamino)phosphine is quickly injected in the above mixture. After the phosphorous precursor injection, the InP nanocrystal synthesis proceeded. The InP core reaction occurs during 3 min. At 3 min, slow injection of 0.6 mL of saturated TOP-S (2.2 M) was done. At 17 min, injection of 1 mL of stoichiometric TOP-S (2.2 M) was done. At 30 min, slow injection of 1 g of Zn(stearate)2 in 4 mL of octadecene was done. At 60 min, temperature is increased from 220 to 240 °C. At 65 min, injection of 0.7 mL of stoichiometric TOP-S (2.2 M) was done. At 90 min, injection of 1 g of Zn(stearate)2 in 4 mL of octadecene was done. At 95 min, temperature is increased from 240 to 260 °C. At 150 min, the reaction ended. At the end of the reaction, the temperature is cooled down. InP/ZnS nanocrystals are then precipitated in ethanol and suspended in chloroform. A 3.2 nm InP core with a 2 nm-thick ZnS shell was prepared. The QY was measured to be 47%.

Then the core/shell QD solutions with very low concentration were spun onto the Si substrate with a speed of 1500 rpm for 30 s. After drying, we measured their emission and find the intensity nearly the same, which avoids the influence of the QDs loading in the films. And then, three samples of them were treated by thermal annealing; the temperatures were set at 180, 200, and 220 °C, respectively with a treatment time of 5 min [18, 19]. The annealing procedure was carried out under a nitrogen atmosphere at ambient pressure using a commercially available RTA reactor (Accu Thermo AW410, Allwin 21 Corp.). For the PL measurement, the emissions of the samples were recorded using LAB-RAM Infinity system. During the measurement, a 488-nm Argon laser was used as the excitation source.

## Results and Discussion

**Fig. 1:**
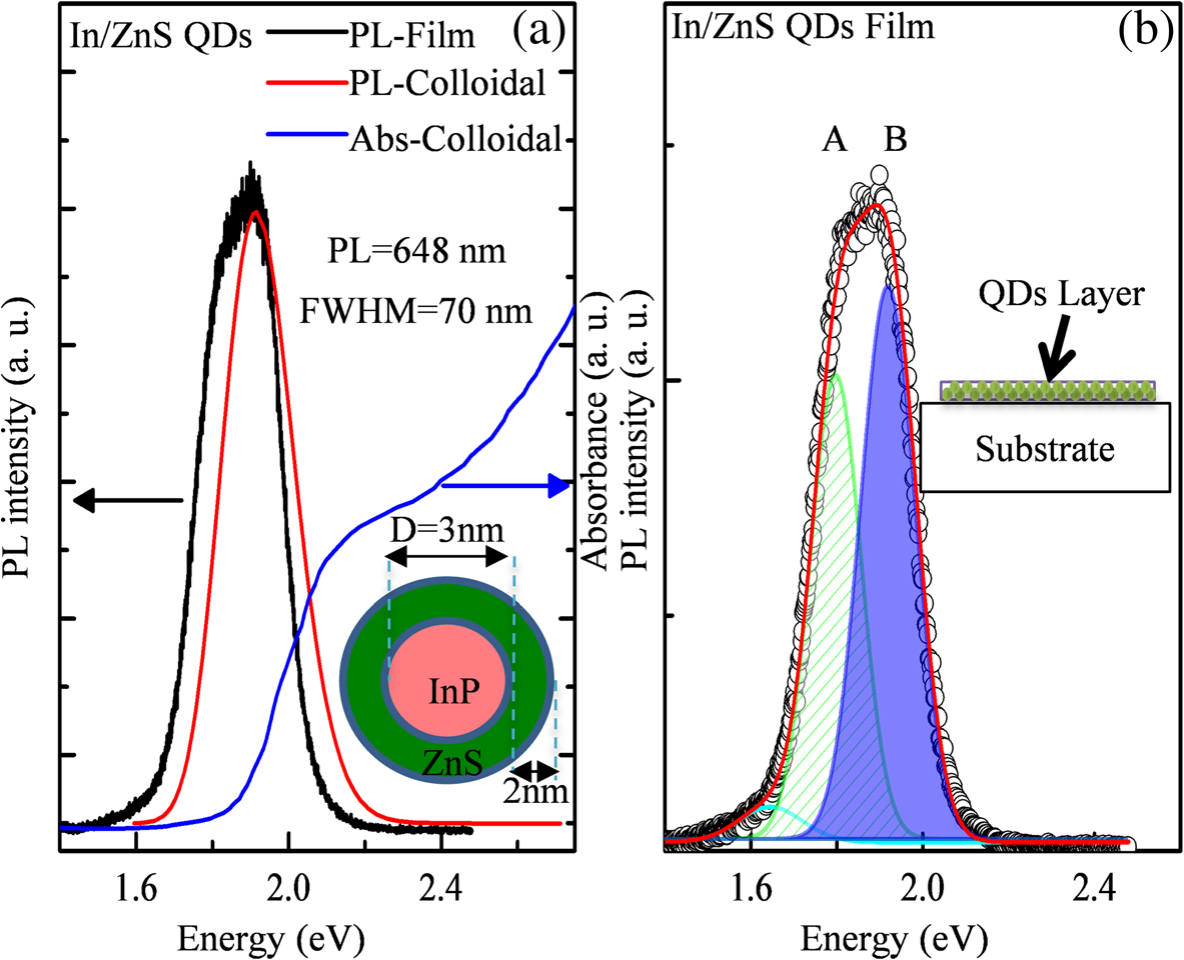
**a** PL spectra of InP/ZnS QD film (untreated) (black line) and QDs in colloidal solution (red line). Absorption spectrum of the colloidal QDs in colloidal solution (blue line). The insert picture is the structure of InP/ZnS core-shell QDs. **b** Peak fitting of PL spectra of InP/ZnS QD film (untreated) (black line). The green and blue are the fitting curve of this spectra, which are named A and B. The insert picture is the structure of InP/ZnS QD films

Figure 1a shows the PL spectra (red line) and absorption spectra (blue line) of colloidal InP/ZnS QDs in solution. The absorption and PL peaks are located at 2.215 eV (560 nm) and 1.914 eV (648 nm), respectively. The FWHM of the PL peak is 70 nm. The black line is the PL spectra of InP/ZnS QD films. Compared with the PL spectra of QDs in solution, a new peak appears at the low energy side. The reason for these differences may be due to the cluster of the QD in film state as reported before [20]. The colloidal QDs in solution are well dispersed and have been protected by surface ligands. Therefore, the colloidal QDs in the solution are relatively stable. As for the QD film, surface ligands will be broken and QDs will be easier to get clusters, introducing a more local state. As shown in Fig. 1b, the PL spectra of InP/ZnS QD film could be well-fitted by three individual Gaussian peaks, namely, the low-energy tail, peak A, and peak B. The low-energy tail is probably due to shallow level defects as described in other articles [21, 22]. Peak A locates at 1.80 eV, with the FWHM of 0.140 eV, while peak B locates at 1.923 eV and the FWHM is 0.151 eV. The origin of peaks A and B will be analyzed through power-dependent PL and temperature dependence photoluminescence (TDPL) later.

**Fig. 2:**
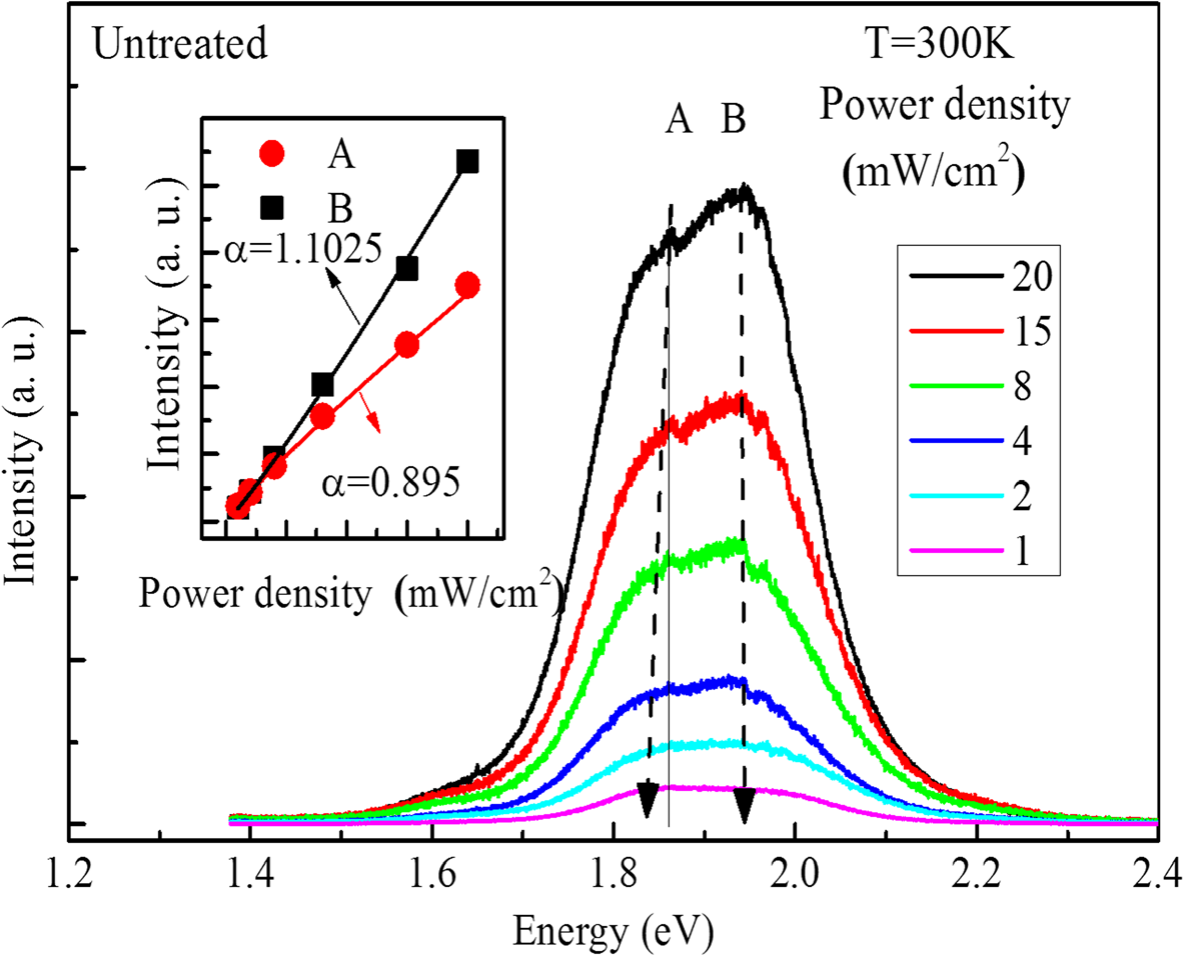
The excitation power-dependent PL spectra of the untreated sample. The insert picture is the integrate intensity of peaks with the change of excitation power; the solid lines are theoretical fitting curves

Figure 2 shows the excitation power-dependent PL spectra of the QD film measured at room temperature. The insert is the integrated PL intensity of the peaks with excitation power. The excitation power-dependent PL intensity is widely used to determine the origin of emission. It has been pointed out that the PL intensity (*I*) can be expressed as the following equation [22, 23],

1 $$ I=\eta {I}_0^{\alpha } $$

where *I*_0_ is the power of the excitation laser, *η* is the emission efficiency, and the exponent *α* represents the radiative recombination mechanism. For recombination of excitons, the value of *α* has been reported to be in the range 1 < *α* < 2. For band to band transition, *α* ≈ 2. For impurity- or defect-related emission, the value of *α* is less than 1, such as free-to-bound recombination and donor–acceptor transition [24–26]. According to the equation, the parameter *α* can be obtained to be 0.895 for peak A and 1.103 for peak B. Moreover, it can be seen that with the increase of excitation power, peak A shows a slight blueshift, which agrees well with the conclusion that peak A comes from the localization states [27]. From the discussions shown above, it can be concluded that peak A is emission related to localized states, and peak B is due to the free-carrier transition.

**Fig. 3:**
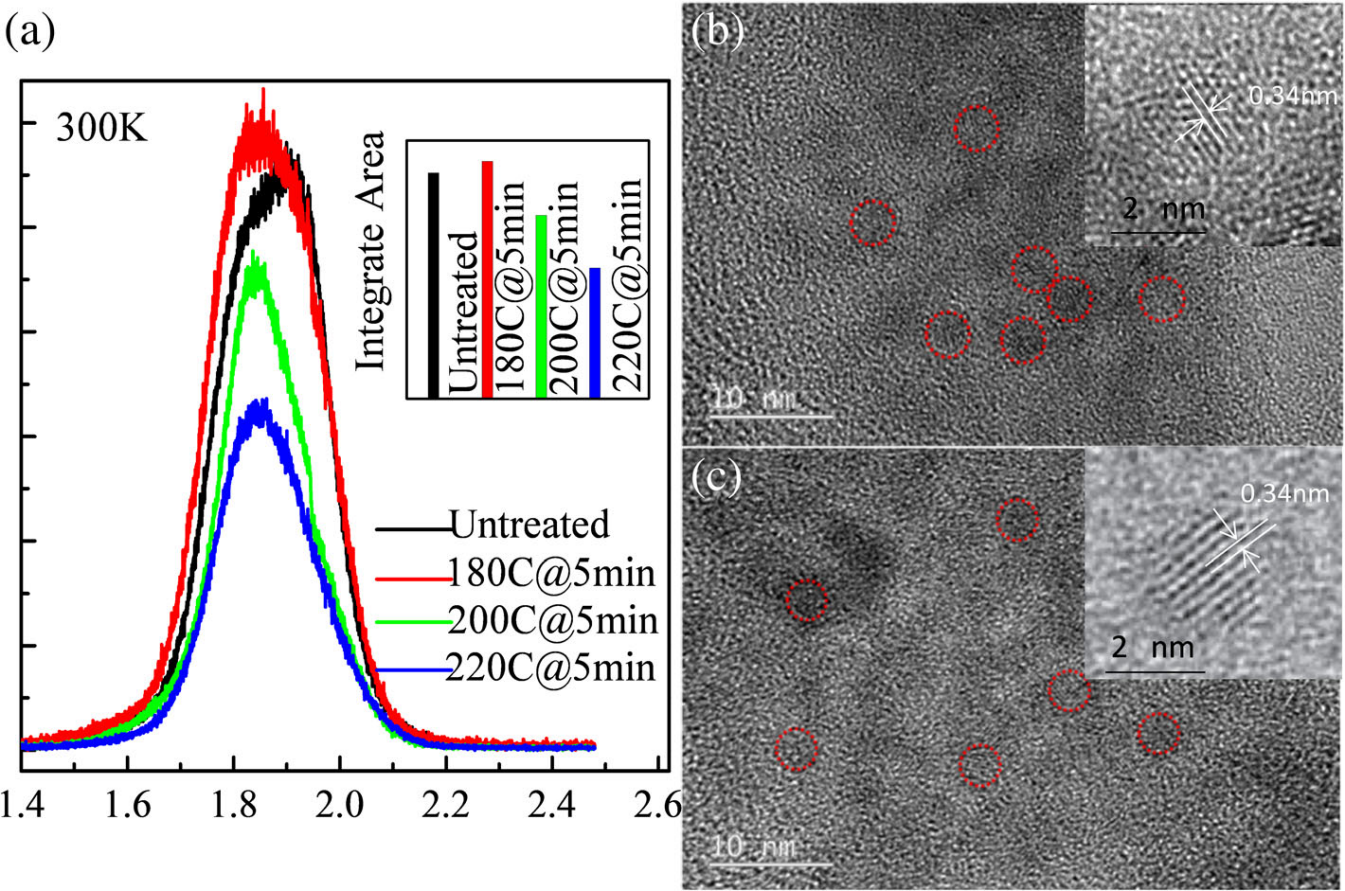
**a** PL spectra of the InP/ZnS QD film (black line) and after annealing at 180 °C (red line), 200 °C (blue line), and 220 °C (cyan line) for 5 min at room temperature. Insert picture shows the integrated intensities of the PL spectra for different samples. The transmission electron microscopy (TEM) images of the untreated sample (**b**) and sample annealed at 180 °C for 5 min (**c**)

The PL spectra of all the four samples at room temperature are displayed in Fig. 3a. After annealing at 180 °C, the absolute intensities of the PL spectra increased. The intensity of the emission contrarily decreases with the further increase of annealing temperature, which has been shown in the inset of Fig. 3a. Later, the untreated sample and the sample annealed at 180 °C for 5 min will be discussed intensively. For the sample annealed at 200 and 220 °C, the annealing process introduced other non-radiative recombination centers, suppressing the free carrier emission. Figure 3b, c displays the transmission electron microscopy (TEM) images of the untreated sample and sample annealed at 180 °C for 5 min, respectively. From these two images, the same QDs’ shape, size, and crystalline can be found. It can be seen from the TEM image that the lattice constant is 0.34 nm, which is consistent with the (111) crystal plane of the sphalerite structure InP, and the core size is about 3 nm.

**Fig. 4:**
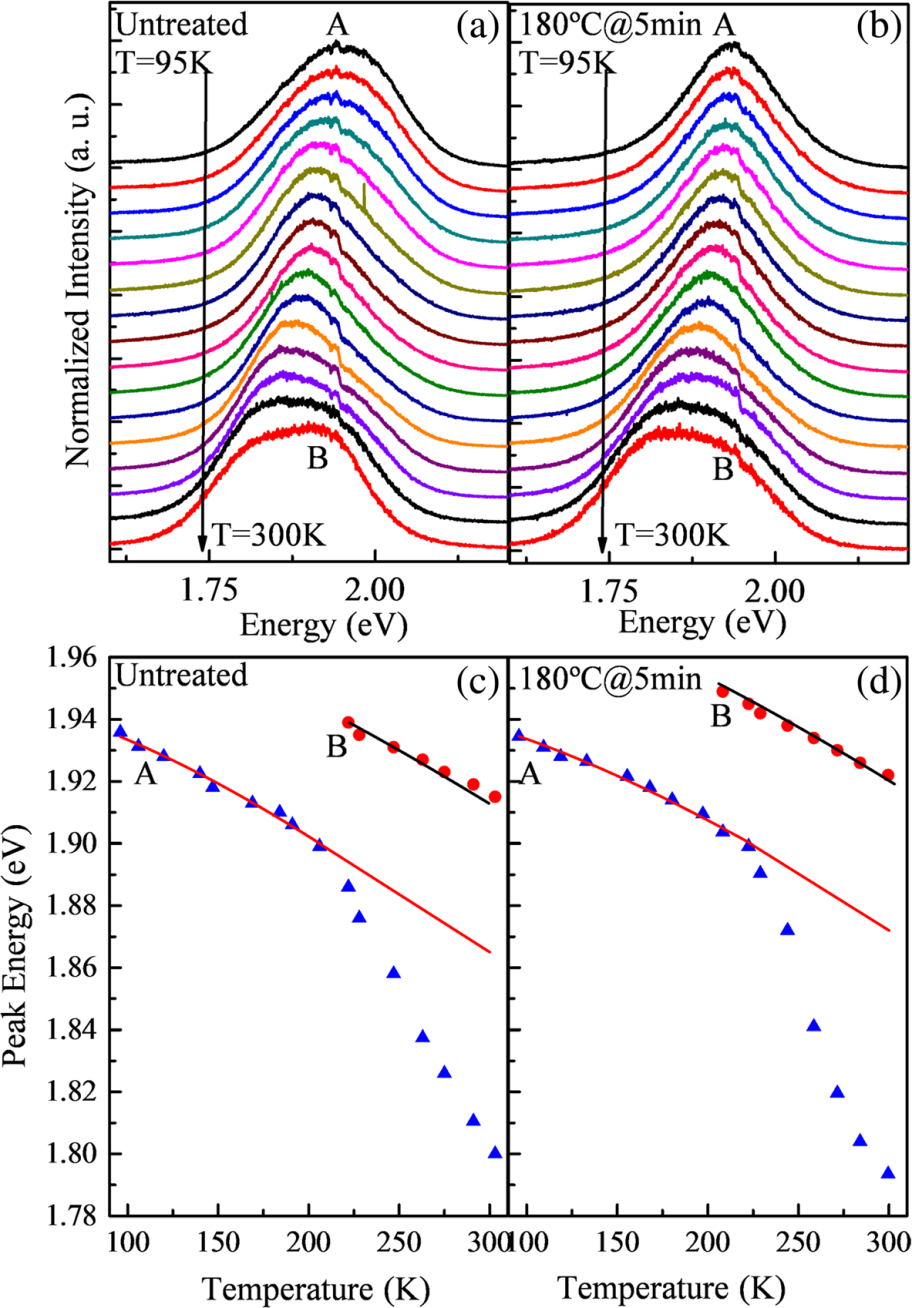
TDPL of untreated sample (**a**) and sample annealed at 180 °C for 5 min (**b**). Peak position of the A and B spectra components from InP/ZnS QDs as a function of temperature for the untreated sample (**c**) and sample annealed at 180 °C for 5 min (**d**). Dots are experimental data. Lines are the fits with Varshni’s equation

The TDPL measurements of two samples have been conducted, as shown in Fig. 4a, b. Figure 4c, d shows the PL peak position as a function of temperature for the two samples. Solid lines are the fitting curves according to Varshni’s equation [28], which gives the temperature dependence of the band gap of bulk semiconductors and has also been used for quantum dots [21, 22, 29, 30],

2 $$ {E}_g(T)={E}_g(0)-\frac{\alpha {T}^2}{\beta +T} $$

where *E*_*g*_(0) is the band gap at 0 K, *α* is the temperature coefficient, and the value of *β* is the Debye temperature. From the graph, it can be seen that peak B can be well fitted by Varshni’s equation, which suggests peak B emissions from the near band emission. The parameters obtained from the fitting are *E*_*g*_(0) = 1.983   eV, *α* = 4.910 × 10^−4^   eV/K, and *β* = 320   K for the untreated sample and *E*_*g*_(0) = 1.991   eV, *α* = 4.896 × 10^−4^   eV/K, and *β* = 320   K for the annealed sample. It can be seen that the values of *α* and *β* are nearly the same as those of bulk InP, which are *α* = 4.91 × 10^−4^   eV/K and *β* = 327   K [31]. For *E*_*g*_(0), it has an 8-meV blueshift after annealing. This is probably due to the inter-diffusion of atoms near the core-shell interface and leads to the decrease of InP core. For peak A, it can be fitted well from 95 to 200 K. After 200 K, it shows an increasing red shift according to Varshni’s equation. This phenomenon can be explained by the carrier’s localization. It is known that with the increase of temperature, the carriers from the shallow level defect states will gain enough energy to escape and become free carriers. From the experimental results, it can be seen that both samples show a carrier localization effect. However, after annealing, the depth of localization states increased. From the fitting curve, we can conclude that peak A originates from the localization states, and peak B emissions from the free carrier transition. This result is in accordance with the result from the excitation power-dependent PL spectra of the untreated sample.

**Fig. 5:**
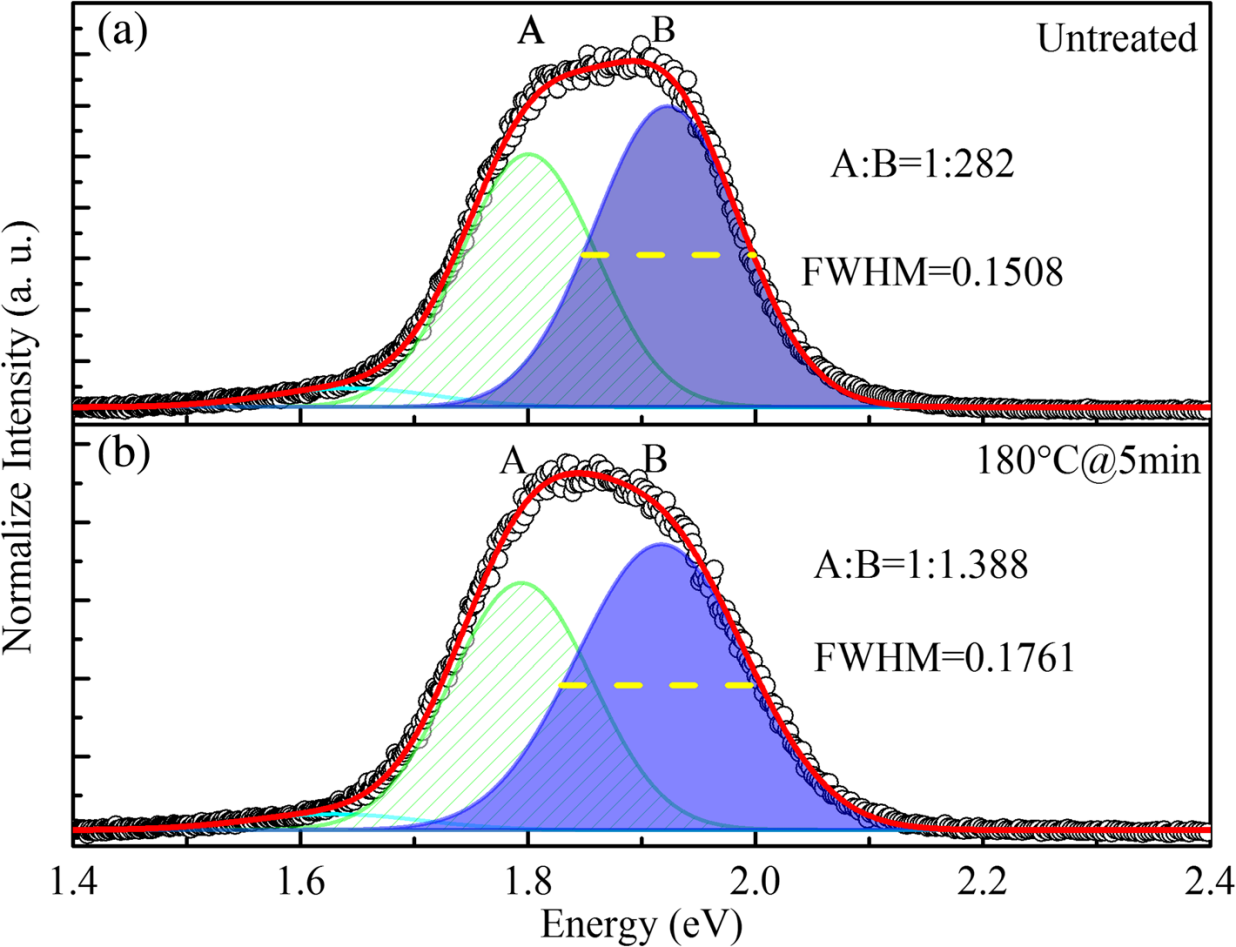
The fitted curves by three individual Gaussian peaks of two samples (**a** for untreated sample and **b** for sample annealed at 180 °C for 5 min) in 300 K

From Fig. 5a, b, we can clearly see the increase of free carrier emission, which can be interpreted by the removal of some shallow localization states of QDs after annealing. Compared with the PL spectra of these two samples at 300 K, we found that, for the untreated sample, peak A and B emissions are located at 1.798 and 1.917 eV, respectively. For the sample annealed at 180 °C for 5 min, the A and B emissions are located at 1.794 and 1.922 eV, respectively. After annealing, the peak positions are nearly the same, but the FWHM of peak B broadens from 0.1508 to 0.1761 eV.

**Fig. 6:**
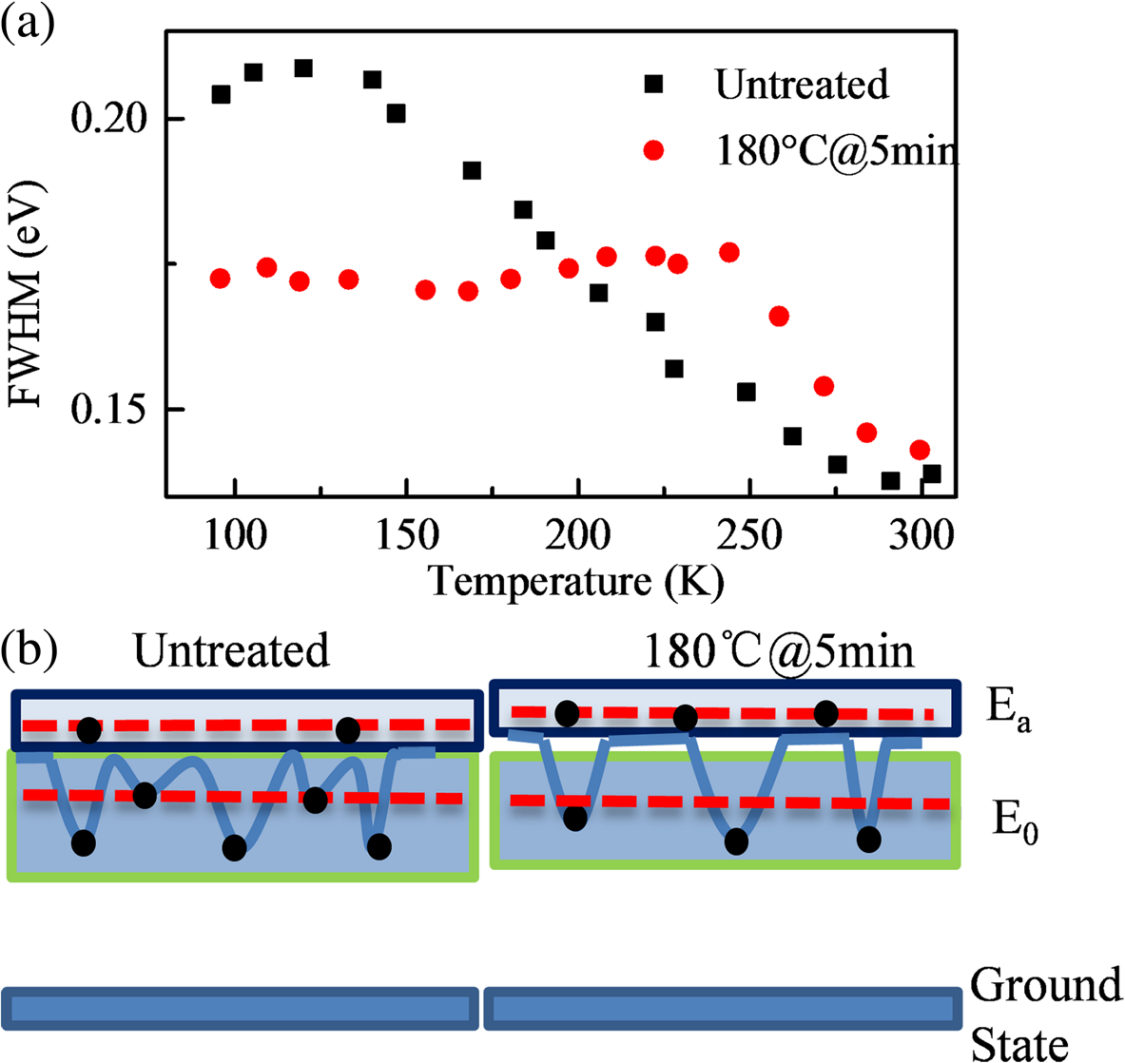
**a** The FWHM changes with temperature of peak A for the untreated sample and sample annealed at 180 °C for 5 min, respectively. **b** The change of energy band by annealing on QD films

Figure 6a shows the FWHM variation with temperature of peak A for the untreated sample and sample annealed at 180 °C for 5 min. For the untreated sample, the FWHM of peak A decreases with increasing temperature. However, for the sample annealed at 180 °C for 5 min, the FWHM is nearly the same before 250 K and then narrows down with increasing temperature. In the usual cases, the FWHM of TDPL spectra will homogeneously broaden with increasing temperature because of the scattering of the exciton by acoustic and optical phonons [32]. For better understanding, an energy band diagram after annealing is used to study the phenomenon and fitting curve of peak A in Fig. 4, as shown in Fig. 6b. There are a series of localization states in the samples to form peak A. When the temperature increases, the carriers at shallow localization states can escape, causing the red shift compared with Varshni’s equation and the narrowing down of FWHM. When the thermal annealing is performed, some shallow localization states are removed. So the FWHM were constant and then narrowed down.

## Conclusions

In summary, we have reported the enhancement of optical properties of thermal annealing on InP/ZnS core/shell QD films. By combining temperature-dependent emission peak position and power dependence spectra, we find direct evidence that peak A emission is from localization states and peak B from free-carrier emission. Referring to the energy band structure revealed by TDPL spectrum, the change of temperature-dependent emission peak position is quantitatively described based on thermal activated redistribution of localized excitons. With the discussion of the effect of annealing on PL spectra, we find annealing significantly increases the emission of free carrier for the removal of some localization states. Our studies have significance on the application of QD devices in electroluminescence or down-conversion of light-emitting applications.

## Abbreviations

FWHM: Full width at half maximum
PL: Photoluminescence
QDs: Quantum dots
QY: Quantum yield
RTA: Rapid thermal annealing
TDPL: Temperature dependence photoluminescence
TEM: Transmission electron microscopy
